# Enzyme kinetic and binding studies identify determinants of specificity for the immunomodulatory enzyme ScpA, a C5a inactivating bacterial protease

**DOI:** 10.1016/j.csbj.2021.04.024

**Published:** 2021-04-17

**Authors:** Malgorzata Teçza, Todd F. Kagawa, Monica Jain, Jakki C. Cooney

**Affiliations:** a Department of Biological Sciences, University of Limerick, Limerick, Ireland; b Department of Chemical Sciences, University of Limerick, Limerick, Ireland; c Bernal Institute, University of Limerick, Limerick, Ireland; d SSPC, University of Limerick, Ireland

**Keywords:** Complement factor C5a, C5a peptidase, COVID-19, cell envelope protease, Immune modulation, Substrate specificity

## Abstract

The Streptococcal C5a peptidase (ScpA) specifically inactivates the human complement factor hC5a, a potent anaphylatoxin recently identified as a therapeutic target for treatment of COVID-19 infections. Biologics used to modulate hC5a are predominantly monoclonal antibodies. Here we present data to support an alternative therapeutic approach based on the specific inactivation of hC5a by ScpA in studies using recombinant hC5a (rhC5a). Initial characterization of ScpA confirmed activity in human serum and against rhC5a desArg (rhC5a_dR_), the predominant hC5a form in blood. A new FRET based enzyme assay showed that ScpA cleaved rhC5a at near physiological concentrations (*K*_m_ 185 nM). Surface Plasmon Resonance (SPR) and Isothermal Titration Calorimetry (ITC) studies established a high affinity ScpA-rhC5a interaction (*K*_D_ 34 nM, $K_{D}^{\mathrm{ITC}}$ 30.8 nM). SPR analyses also showed that substrate binding is dominated (88% of ΔG°_bind_) by interactions with the bulky N-ter cleavage product (P_N_, ’core’ residues 1–67) with interactions involving the C-ter R74 contributing most of the remaining ΔG°_bind_. Furthermore, reduced binding affinity following mutation of a subset of positively charged Arginine residues of P_N_ and in the presence of higher salt concentrations, highlighted the importance of electrostatic interactions. These data provide the first in-depth study of the ScpA-C5a interaction and indicate that ScpA’s ability to efficiently cleave physiological concentrations of C5a is driven by electrostatic interactions between an exosite on the enzyme and the ‘core’ of C5a. The results and methods described herein will facilitate engineering of ScpA to enhance its potential as a therapeutic for excessive immune response to infectious disease.

## Highlights

• The human complement protein C5a is implicated in immunomodulatory diseases.
• ScpA, a C5a inactivating protease, represents a novel enzymatic approach to therapy.
• High-affinity ScpA specificity for C5a is driven by C5a core-exosite interactions.
• 3 Arginines in the C5a core, and electrostatic interactions contribute to binding.
• These studies are first steps in the development of novel immunomodulatory therapies.

## Graphical abstract

**Figure:**
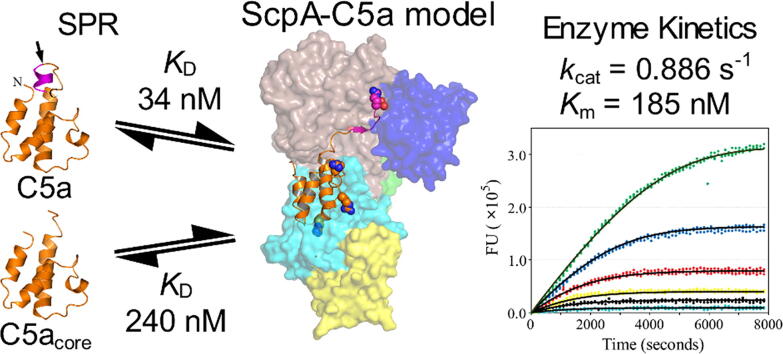

## 1 Introduction

The role of the complement factor C5a in inflammatory diseases has come into sharp focus in recent months, with data supporting a role for C5a-C5aR in the progression of COVID-19 disease [1]. The therapeutic value of targeting C5a as an intervention in COVID-19 infection is demonstrated by multiple ongoing Phase III clinical studies using anti-C5a monoclonal antibodies (MAbs) [2], [3], [4], [5]. Complement therapeutics have garnered increasing attention as strategies for treatments of inflammatory diseases [6], [7]. Proteases are also being explored for their potential to modulate the complement response in the disease process. Examples include an engineered human MTSP1 protease targeting C3 as a treatment of age-related macular degeneration (AMD) [8] and the use of ScpA, a bacterial protease specific for C5a, as an intervention in sepsis [9].

The ScpA enzyme is one of two multi-domain cell envelope proteases (CEPs) produced by the Gram-positive bacterium *Streptococcus pyogenes*. The second enzyme is the IL-8 cleaving peptidase ScpC (also known as SpyCEP). Unlike degradative subtilases, ScpA and ScpC have high substrate specificity. ScpC has been shown to selectively cleave and inactivate CXC chemokines that possess the ELR motif [10], thus targeting a structurally conserved carboxyl-terminal (C-ter) helix in chemokines which stimulate neutrophil migration [11], [12]. ScpA is a more selective enzyme specific for the complement factors C5a and C3a only [44] [13], [14]. The better characterized substrate for ScpA is C5a, a 74 amino-acid pro-inflammatory product of complement activation with a myriad of significant biological functions [13], [15]. ScpA inactivation of human C5a (hC5a) results from a single proteolytic event between residues H67 and K68 [13], releasing the 7 C-terminal (C-ter) residues of the chemotaxin associated with receptor activation [16]. The amino-terminal (N-ter) cleavage product (P_N_, residues 1–67, referred to as the ‘core’ residues or ‘hC5a_core_’) has greatly reduced PMN chemotactic and stimulatory activity as compared to the full-length C5a peptide [17]. From the perspective of bacterial virulence, the action of these two CEPs modulate the host immune response and promote bacterial persistence.

The current study examines factors contributing to ScpA’s specificity for hC5a, and therefore how it exerts an immune-modulatory effect. Early characterizations of the peptidase showed that ScpA did not cleave native forms of a variety of proteins (C5, human serum albumin, ovalbumin, soybean trypsin inhibitor, carbonic anhydrase, α-lactalbumin, myosin, and cytochrome), but demonstrated endoproteolytic activity against hC5a [13], [15]. More recently, ScpA has also been shown to be capable of cleaving C3a, the structurally related complement derived anaphylotoxin [14]. In addition, ScpA was observed to discriminate between C5a molecules from different species, inactivating bovine C5a but not the mouse or rat proteins [18].

While the specificity of ScpA has not been exhaustively tested, it has historically been considered a highly discriminating enzyme. The specificity of ScpA has been examined in both enzyme kinetic and substrate binding. To date, enzyme kinetic studies on ScpA have relied on use of small peptide mimics or large chimeric fusion proteins as substrate analogues. Studies with a 16 amino-acid peptide representing the C-ter of hC5a, reported *k*_cat_ and *K*_m_ values of 1.1 s^−1^ and 360 μM respectively for ScpA [19]. Additional analysis with a peptide substrate lacking the 7 C-ter tail residues decreased *k*_cat_/*K*_m_ more than 200 fold, demonstrating the importance of prime side interactions in determining substrate interactions. Several studies expanded the substrate to include the entire C5a molecule. Stafslien and Cleary employed a Glutathione S transferase-C5a-green fluorescent protein tripartite fusion as a substrate and reported specific activity for ScpA, but not other kinetic parameters [20]. However, their study did confirm the identity of the catalytic residues (D130, H193, and S512), and demonstrated that peptidase activity decreased with increasing (20–200 mM) NaCl concentration [20]. In addition, Terao and co-workers examined interaction between active ScpA and the full-length human C5a in Surface Plasmon Resonance (SPR) studies which indicated that the peptidase binds hC5a with very low affinity (*K*_D_ 7.25 mM) [21].

When considering activity of an enzyme such as a protease, it is important to relate the observations on *K*_m_ to the physiological concentration of its substrate. Data from clinical samples under conditions where C5a is expected to be elevated indicate that physiological levels of C5a are in the low nM range (10–30 nM) for a variety of clinical indications including intra-abdominal infections [22], sepsis [23] and COVID-19 infection [1], [24]. Thus, the substrate analogues used in previous studies do not adequately account for interactions critical for examining the properties of ScpA. Understanding of ScpA as a virulence factor and potential therapeutic would benefit from an improved substrate for kinetic analysis.

The structure of ScpA showed that it is a subtilisin-like protease with a PA domain inserted within the catalytic domain and 3 tandemly arranged C-terminal fibronectin type III domains (Fn1-Fn3) [44] . Based on proximity to the active site, the PA and Fn2 domains were proposed to participate in substrate interactions in the active site and at an exosite respectively. Furthermore, modelling of the ScpA-hC5a complex indicated that the catalytic site is occluded and would be inaccessible to large, folded substrates. Therefore, positioning of the scissile bond near the catalytic site required that the hC5a residues in the C-ter α-helix (residues 65–74) adopt an extended conformation. Interactions with the Fn2 domain were predicted to make the largest contribution to substrate binding affinity, involving a large surface area and 9 salt bridges with hC5a residues between residue 1 and residue 64. These exosite interactions between the hC5a_core_ residues and the Fn2 domain would compensate for the limited number of stabilizing interactions in non-prime side of the active site. The model also proposed that ionic interactions with 3 hC5a residues in the C-ter tail (K68, D69 and R74) would be involved in substrate binding.

Here we present enzyme kinetic data, along with the surface (SPR) and solution (Isothermal Titration Calorimetry) protein–protein interaction studies that probe the ScpA-hC5a interaction. The results of these studies are the first to be consistent with physiologically relevant concentrations of C5a. These data thus provide in-depth insight into the determinants of ScpA specificity, a critical initial step in developing this enzyme into novel therapeutics for immunomodulatory disorders involving C5a.

## 2 Materials and methods

### 2.1 Preparation of recombinant proteins

The cloning of recombinant ScpA, residues 31–1032, has been described previously [44], and was designed to closely mimic the form of ScpA released from the surface of the Group A Streptococcal cell by the action of SpeB, and which is reported to retain activity [25]. For the purposes of this work this protein is called ScpA. The plasmid expressing ScpA (pGEX ScpA_(31-1032)_) was used as a template to generate an active site serine to alanine (S512A) mutation with the QuickChange II site directed mutagenesis kit (Strategene, USA) for use in SPR studies. The proper folding of ScpA_S512A_ was confirmed crystallographically (Supplemental Information, Table S2 and Fig. S4), and the coordinates and structure factors deposited at the Protein Data Bank (PDB ID 7BJ3).

Recombinant human C5a peptides were produced as N-ter *hexa*-histidine tagged (HT) fusion proteins using a protocol which closely followed the method of Bubeck *et al.* [26]. Synthetic genes for human C5a were inserted into the pProExHTb expression vector (Invitrogen, UK). Unless otherwise stated point mutations were generated using a QuickChange II site directed mutagenesis kit (Strategene, USA) with specific primers, and using the pProEXHTb construct for rhC5a as the template. The synthetic gene for rhC5a with an additional C-ter C75 residue (rhC5a_C75_) was generated using *gblock* technology (IDT Biotech). The rhC5a_core_ was purified from a treatment of rhC5a with ScpA. For competitive binding experiments, TEV protease (Sigma Merck, U.S.A) was used to produce the His-tagless form of rhC5a (rhC5a_-HT_).

Additional details of recombinant protein production and purification are provided in Supplemental Information.

### 2.2 ScpA activity assays

The activity of recombinant ScpA against rhC5a, rhC5a_dR_ and rhC5a_C75_-BODIPY was examined in cleavage assays containing 18 μM C5a peptide and 10 nM ScpA in PBS. The reactions were incubated at 37 °C for 15 min and analyzed by SDS-PAGE and MS. Salt dependent activity assays were conducted in 30 mM Hepes buffer with 10 mM, 150 mM and 1 M NaCl adjusted to pH 7 with KOH. 17 µM rhC5a was cleaved with 10 nM ScpA under each buffering condition for 7 min at 37 °C and assessed with SDS-PAGE.

ScpA activity was also demonstrated in human serum. Reactions were in normal human serum (Merck, Germany) with 39 µM rhC5a and 36 nM ScpA. Assays were incubated for 90 min at 37 °C. The reactions were immediately passed through a ProteoSpin™ protein depletion column (Norgen Biotek, Canada) and rhC5a cleavage assessed with SDS-PAGE. These assays were conducted at higher concentrations of rhC5a to facilitate visualization of the digestion product anticipating dilution of rhC5a using the ProteoSpin™ protein depletion column. Western blot analysis of the samples used polyclonal rabbit anti-human C5a antibody (MBS524143, MyBioSource, USA).

The rate of reaction of cleavage of C5a by ScpA was determined in an assay using the self-quenched doubly BODIPY labelled His-tagged rhC5a_C75_ as a substrate. Production of the substrate is described in Supplemental Information. Enzyme kinetics assays contained 16, 31, 62, 125, 250 or 500 nM of the labelled substrate and 180 pM ScpA in 1 X PBS with 0.1% (v/v) Tween 20. The reactions were performed at 25 °C, and the evolution of fluorescence monitored over 8000 s using a Berthold Tristar2S fluorescence plate reader (Berthold Technologies, UK) with excitation at 485 nm and emission at 520 nm and a 2 s data acquisition time. Experiments were performed in quadruplicate. Following correction for spontaneous hydrolysis of substrate the progress curves were analysed using the DYNAFIT software package [27]. Progress curves were fit with the minimal Van Slyke-Cullen mechanism [28] to obtain steady state enzyme kinetic parameters (*K*_m_ and *k*_cat_).

### 2.3 Surface plasmon resonance studies

SPR data was obtained by measuring of the His-tagged C5a peptides to ScpA_S512A_ was measured at 25 °C with a BIAcore X100 system (GE Healthcare, UK) in Hepes buffer (10 mM Hepes-KOH pH 7.5, 150 mM NaCl, 0.005% (v/v) Tween 20, 50 μM EDTA). The experiments were conducted in triplicate. Values of binding constants reported on Table 1 represent the mean and standard deviation from three experiments. Interactions with the human C5a peptides (rhC5a, rhC5a_dR_, rhC5a_core_ and point mutations) were assessed with an NTA sensor chip (GE Healthcare, UK) with approximately 30–40 response units (RU) of immobilized peptide ligand and a flow rate of 30 μL/min. The binding to human C5a peptides was measured over a range of ScpA_S512A_ concentrations (32-fold) divided equally over 6 experiments (*i.e.* 2-fold change in ScpA_S512A_ concentration between measurements). Association and dissociation phases were each monitored for 200 s. The highest ScpA_S512A_ concentrations were 90, 500, and 1000 nM for rhC5a, rhC5a_dR_, and rhC5a_core_ binding respectively. The highest ScpA_S512A_ concentration was 90 nM for rhC5a_K4A,K5A_, rhC5a_K12A,K14A_, and rhC5a_K49A_, 360 nM for rhC5a_R46A_, and 720 nM for rhC5a_R37A_ and rhC5a_R40A_. Double referencing was used to remove the effects associated with buffer changes. In addition, the signal associated with non-specific ScpA_S512A_ interactions with the chip surface was subtracted from each sensorgram. The sensorgrams were fit in a global analysis with the BIAevaluation 4.1 curve fitting software (GE Healthcare, UK). The best fit (lowest chi^2^) was obtained using 1:1 Langmuir model with a drifting baseline.

**Table 1:** Kinetic and thermodynamic parameters for ScpA_S512A_ binding to C5a peptides.

	*k*_a_ × 10^4^ (M^−1^s^−1^)^a^	*k*_d_ × 10^−3^ (s^−1^)^a^	*K*_D_ (nM)*^a^*	ΔΔG° (kcal/mol)*^b^*
rhC5a (150 mM NaCl)	13.1 ± 0.3	4.4 ± 0.3	34 ± 3	0*^c^*
rhC5a (350 mM NaCl)	6.85 ± 0.95	4.31 ± 0.28	63.5 ± 7.5	0.4
rhC5a (550 mM NaCl)	3.92 ± 0.38	5.4 ± 0.5	140 ± 20	0.8
rhC5a (750 mM NaCl)	2.56 ± 0.28	6.51 ± 0.43	256 ± 30	1.2
rhC5a (950 mM NaCl)	1.953 ± 0.137	9.10 ± 0.78	466 ± 30	1.5
rhC5a_core_	3.52 ± 0.05	8.5 ± 0.3	240 ± 10	1.2
rhC5a_dR_	6.17 ± 0.52	11.8 ± 1.0	191 ± 9	1.0
rhC5a_K4A,K5A_	18.0 ± 1.2	5.83 ± 0.37	32.5 ± 3.0	0.0
rhC5a_K12A,K14A_	15.8 ± 1.2	4.05 ± 0.30	25.6 ± 0.3	−0.2
rhC5a_R37A_	8.12 ± 0.49	17.6 ± 0.1	217 ± 11	1.1
rhC5a_R40A_	8.80 ± 0.74	20.5 ± 1.4	234 ± 20	1.1
rhC5a_R46A_	11.1 ± 1.3	17.8 ± 0.7	162 ± 21	0.9
hC5a_K49A_	19.5 ± 1.0	5.0 ± 0.7	26 ± 2	−0.2

*a* Reported as mean and standard deviation from 3 experiments.

*b* ΔΔG° = −RTln[*K*_D_ /*K*_D_ (rhC5a, 150 mM NaCl)], R = 1.986 (cal mol^−1^ K^−1^), T = 298 K.

*c* Binding energy for rhC5a at 298 K , ΔG°_bind_ = -RTln[1/*K*_D_] = -10.2 kcal/mol.

### 2.4 Competitive binding assay

**Scheme 1:**
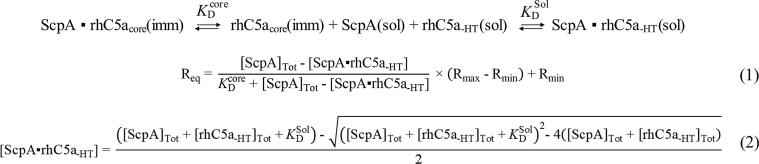
The scheme shows the equilibria involved in the competition between ScpA binding to full length rhC5a in solution vs rhC5a_core_ immobilized to the chip surface (rhC5a_-HT_(sol) and rhC5a_core_(imm) respectively). The equilibrium dissociation constant for ScpA-rhC5a_-HT_ binding in solution ($K_{D}^{\mathrm{Sol}}$) is obtained by fitting of equilibrium response units (R_eq_) measured with varying concentrations of rhC5a_-HT_(sol) using Equation 1. [ScpA▪rhC5a_-HT_] in Eq. 1 is obtained from the quadratic solution for the expression for KDsol (Eq. 2). $K_{D}^{\mathrm{core}}$ is the equilibrium dissociation constant for ScpA binding to immobilized rhC5a_core_ obtained from SPR experiments (Table 1).

The competition between ScpA binding to immobilized rhC5a_core_ and the His-tagless form of rhC5a in solution (rhC5a_core_(imm) and rhC5a_-HT_(sol) respectively in Scheme 1) was used to estimate the ScpA-rhC5a equilibrium dissociation constant in solution ($K_{D}^{\mathrm{Sol}}$). The equilibrium SPR response (R_eq_) was measured over a range rhC5a_-HT_ concentrations with fixed levels of immobilized rhC5a_core_ and ScpA concentrations. 40 RU of HT-rhC5a_core_ was immobilized on a NTA biosensor chip. Mixtures of ScpA_S512A_ (700 nM) and rhC5a_-HT_ (0–2000 nM) were pre-incubated in SPR running buffer (1 hr on ice) prior to injection over both flow cells (30 µL/min for 180 sec). R_eq_ values obtained from these experiments were analyzed according to the method reported by Morelock *et al.* [29].

In brief, the R_eq_ in the competition assay measures ScpA_S512A_-rhC5a_core_ binding on the chip surface, which is dependent on the concentration of free ScpA_S512A_ in solution (*i.e.* [ScpA]_Tot_ – [ScpA▪rhC5a_-HT_] (Eq. 1). Where [ScpA]_Tot_ is the total concentration of ScpA_S512A_, [ScpA▪rhC5a_-HT_] is the concentration of the ScpA_S512A_▪rhC5a_-HT_ complex in solution, $K_{D}^{\mathrm{core}}$ is the SPR derived constant for ScpA-rhC5a_core_ binding (Table 1), and R_max_ and R_min_ are the maximum and minimum R_eq_ responses in the competition assay. [ScpA▪rhC5a_-HT_] can be obtained from the quadratic solution of the expression for $K_{D}^{\mathrm{Sol}}$ (Eq. 2). Substitution of Eq. 2 into Eq. 1 allows estimation of $K_{D}^{\mathrm{Sol}}$ by fitting plots of [rhC5a_-HT_]_Tot_ versus R_eq_ with fixed values of [ScpA]_Tot_ (700 nM), $K_{D}^{\mathrm{core}}$ (240 nM), and R_min_ (0.0).

### 2.5 ITC determination of equilibrium dissociation constant ($K_{D}^{\mathrm{ITC}}$)

Isothermal titration calorimetry (ITC) was used to measure thermodynamic parameters for ScpA_S512A_-rhC5a binding in solution. Measurements were performed using an iTC_200_ calorimeter (MicroCal, U.S.A.) equipped with a 200 μL sample cell (GE Healthcare). Purified protein samples were extensively dialysed against 50 mM Hepes-KOH buffer, pH 7.5 containing 150 mM NaCl. The reference power was set to 4.5 μcal/sec and the instrument was equilibrated to 25 °C. ScpA_S512A_ (18 μM) was placed in the sample cell and titrated with 170 μM rhC5a in 2.5 μL injections with 120 s interval between the injections and stirring (1000 rpm). Titration data were collected in triplicate and analysed with the ORIGIN software package (MicroCal, U.S.A.). The peaks areas were integrated and corrected for the heat of rhC5a dilution. Plots of kcal/mol of injectant versus molar ratio were analyzed with the ‘one set of sites’ model to obtain values for the association binding constant (*K*_A_), stoichiometry (N), and the enthalpy of binding (ΔH_bind_), entropy of binding (ΔS_bind_) were determined. The reported equilibrium dissociation constant ($K_{D}^{\mathrm{ITC}}$) was derived from *K*_A_.

### 2.6 Mass spectrometry analysis of recombinant rhC5a and rhC5a_C75_ proteins

Intact mass analysis of rhC5a and rhC5a_C75_ proteins were performed in-house on a Bruker UltrafleXtreme instrument (Bruker Daltonik GmbH, Germany), using Compass 1.4 software unless otherwise stated. The sample preparation followed the dry droplet, two-layer method as per [30]. Protein samples were diluted with 0.1% TFA to final concentrations of 50 µg/mL and 15 µg/mL for rhC5a and rhC5a_C75_ samples, respectively. Analyte solutions consist of a 1:1 mixture of the diluted protein samples and a saturated solution of sinapinic acid (Bruker Daltonik GmbH, Germany) in 30% acetonitrile, 0.1% TFA (v/v). A saturated matrix solution of sinapinic acid in ethanol was layered onto a stainless-steel target plate and allowed to air dry. 0.5 µL of the analyte solution was applied as the second layer to the target and allowed to dry. Spectra were recorded in the positive linear mode and externally calibrated using Protein Calibration Standard I (Bruker Daltonik GmbH, Germany). Germany). Masses of rhC5a and its mutants were calculated from amino acid sequences with the PROTPARAM tool on the ExPASy server [31]. Calculated masses include an additional 70 Da to account for disulfide bond linkages and the addition of β-mercaptoethanol to C27 in the refolding process.

## 3 Results

### 3.1 ScpA is active against both major forms of C5a found in blood

**Fig. 1:**
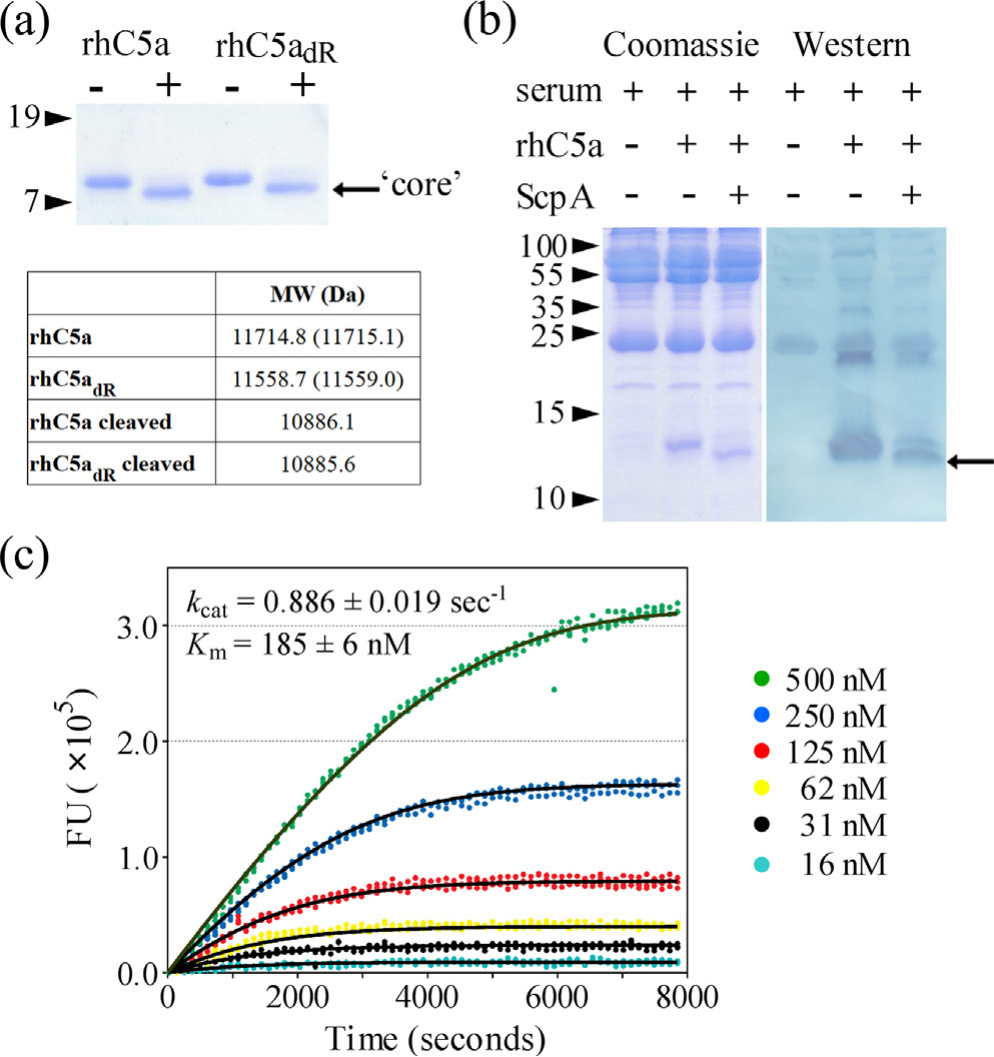
ScpA enzyme kinetics and activity against rhC5a_dR_. (a) SDS-PAGE analysis of recombinant rhC5a and rhC5a_dR_ cleaved with ScpA. Lanes with ‘+’ and ‘-’ indicate samples with and without ScpA, respectively. rhC5a and rhC5a_dR_ treated with ScpA produced fragments of similar size (indicated with an arrow marked ‘core’). Molecular weight ladders are shown on the left side of the gels in Fig. 1a and 1b in kDa. Mass spectrometry of cleaved rhC5a and rhC5a_dR_ show that the observed mass of the products (10886.1 Da and 10885.6 Da, respectively) are consistent with rhC5a_core_ (calculated mass 10886.2 Da.). Observed and calculated (parenthesis) masses are reported for rhC5a and rhC5a_dR_. (b) The activity of ScpA was examined in human serum. The Coomassie stained polyacrylamide gel and Western blot analysis shows that ScpA (36 nM) cleaves rhC5a (39 µM) in human serum. The arrow indicates the expected position for the ‘core’ (P_N_) product of ScpA cleavage of C5a. (c) Progress curves for ScpA cleavage of rhC5a_C75_-BODIPY. Data are plotted with curves from global fitting of 6 progress curves to the Van Slyke-Cullen model. Values for *k*_cat_ and *K*_m_ are reported as the mean and standard deviation of the mean from 3 experiments. Data shown in the plot are from 3 experiments. A key to substrate concentrations is provided on the right-hand side of the graph.

C5a in human blood is rapidly cleaved by carboxypeptidases to the C5a des Arg form which lacks the C-ter R74 residue [32]. While C5a des Arg is reported to have reduced potency as compared to C5a, it still retains biological activity [33], [34]. The ScpA enzyme (defined as residues 32–1032 of the wild-type enzyme) cleaved recombinant forms of human C5a and C5a des Arg (rhC5a and rhC5a_dR_, respectively) in agreement with data on C5a proteins isolated from human serum [15]. A decrease in molecular weight for both forms of C5a was observed in the presence of ScpA (Fig. 1a). The mass of larger digestion product was confirmed to be the core portion of C5a (residues 1–67, rhC5a_core)_ (Fig. 1a). These analyses demonstrated that the same N-ter product (P_N_) was released by ScpA from both rhC5a and rhC5a_dR_. Thus, ScpA is capable of inactivating both major forms of C5a by targeting the same scissile bond between residues H67 and K68. Furthermore, the C5a-ase activity of the ScpA was assessed in a more biologically relevant solution. These assays showed that ScpA readily cleaved rhC5a to near completion in human serum (Fig. 1b), supporting the ability of the recombinant enzyme to function as expected in biological fluids, and demonstrating a potential for the development of ScpA as a therapeutic enzyme.

### 3.2 ScpA cleaves C5a with a K_m_ and k_cat_ of 185 nM and 0.886 *sec*^-1^ respectively

Characterization of the kinetics of C5a cleavage by ScpA utilized fluorescent substrate based on the full length C5a with two self-quenching BODIPY moieties on the core and tail portion of the molecule (Supplemental Fig. S1). The labelling chemistry target the free thiols of the naturally occurring C27 residue and the introduced C-terminal C75 residue (rhC5a_C75_). In preliminary studies, the doubly labelled rhC5a_C75_-BODIPY form of C5a was found to be readily cleaved by ScpA, producing fragments of the expected mass for the labelled P_N_ and tail portions (P_C_) of the substrate (Supplemental Fig. S1).

Kinetic parameters were determined using a full range of substrate concentrations under conditions where the enzyme concentration was much less than the concentration of the substrate (Fig. 1c). Progress curves were fit with the minimal Van Slyke-Cullen mechanism to obtain steady state enzyme kinetic parameters giving a *K*_m_ and *k*_cat_ of 185 ± 6 nM and 0.886 ± 0.019 *sec*^-1^, respectively. The *k*_cat_/*K*_m_ of 4.79 X 10^6^ M^−1^s^−1^ is well below that expected for a diffusion limited enzymatic reaction [35]. In contrast to other substrates used to measure ScpA activity, the observed *K*_m_ for rhC5a_C75_-BODIPY is consistent with the nM concentrations of C5a *in vivo* and thus a very sensitive probe to study the specificity determinants of ScpA.

### 3.3 ScpA binds to rhC5a with nM affinity

**Fig. 2:**
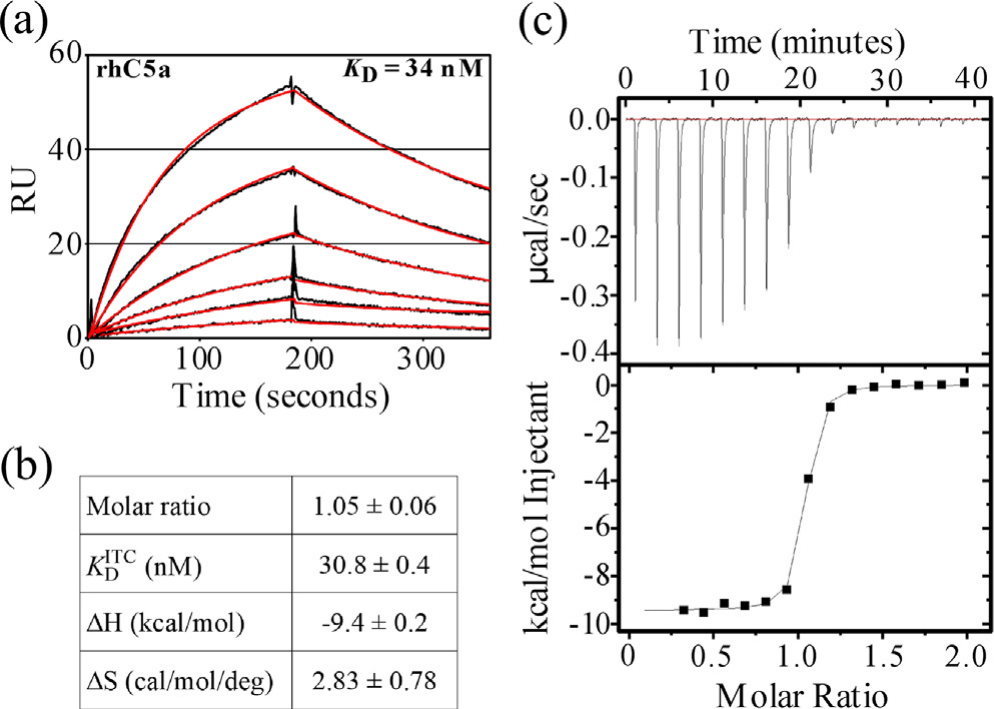
SPR and ITC characterization of ScpA_S512A_-rhC5a binding. (a) Representative SPR sensorgrams of ScpA_S512A_ binding to immobilized full length rhC5a. Observed data (black lines) are shown with curves obtained from global fitting of data with a 1:1 Langmuir model for binding (red lines). The mean *K*_D_ value obtained from 3 experiments is reported. (b) Thermodynamic parameters obtained from calorimetric titration of ScpA_S512A_ with rhC5a. Mean and the standard deviation of the mean are reported for 4 experiments. Panel c shows ITC data from a single measurement. Peaks from the titration data were integrated and corrected for the heat of dilution of rhC5a. The enthalpy per mole of rhC5a injected was plotted against the ScpA_S512A_:rhC5a molar ratio and fit with the ‘one set of sites’ model. (For interpretation of the references to colour in this figure legend, the reader is referred to the web version of this article.)

To explore how ScpA recognizes and binds to its substrate C5a in greater detail, a series of protein–protein interaction studies was performed. To circumvent potential complexities expected when measuring substrate binding to an active enzyme, surface plasmon resonance (SPR) studies utilized the S512A active site mutant of ScpA (ScpA_S512A_) and recombinant His-tagged C5a peptides. The sensorgrams observed for ScpA_S512A_ binding to immobilized rhC5a were well accounted for with global fitting of the data using a 1:1 Langmuir binding model (Fig. 2a). All binding constants are reported on Table 1

For rhC5a binding, the average association (*k*_a_) and dissociation rates (*k*_d_) were 13.1 X 10^4^ M^−1^s^−1^ and 4.4 X 10^-3^ s^−1^ respectively. The equilibrium dissociation constant (*K*_D_) derived from these kinetic parameters was in the low nM range (34 nM) indicating that ScpA has a high affinity for rhC5a. The magnitude of the *k*_a_ is at the theoretical boundary between protein–protein association rates limited by diffusion versus those limited by conformational changes [36]. Faster rates implicate the involvement of complementary long-range electrostatic interactions, while slower rates are expected to result from larger structural rearrangements such as changes in domain orientation.

The SPR data are supported by additional ITC studies (Fig. 2b and c) that more accurately reflect binding in solution. The analysis confirms that the substrate binds to ScpA at a single site with high affinity ($K_{D}^{\mathrm{ITC}}$ = 30.8 nM). The ITC measurements also indicated that the binding of the substrate is an enthalpically driven process with a ΔH of −9.4 kcal/mol and ΔS of 2.3 cal/mol/deg. This is consistent with the published model which highlighted 22 hydrogen bonds, and 15 salt-bridges between the substrate and enzyme [44]. Importantly, the *K*_D_ values reported here indicate that the enzyme is capable of binding C5a at physiological concentrations and is in good agreement with the *K*_m_ measured with the doubly labelled C5a substrate (Section 3.2). The observed values indicate a considerably tighter binding than has previously been reported. The interaction between wild type ScpA and the full-length substrate was examined by others in SPR studies which indicated that the peptidase binds rhC5a with very low affinity (*K*_D_ 7.25 mM) [21]. The discrepancy may be related to differences in the SPR experimental details, most notably the use of the active enzyme which complicates the interpretation of binding data. Taken together these data indicate a high affinity interaction between ScpA and its substrate C5a, and that the described enzyme kinetic and SPR methods are sensitive tools for detailed characterization of ScpA substrate interactions.

### 3.4 Binding studies indicate exosite binding interaction between ScpA and C5a

**Fig. 3:**
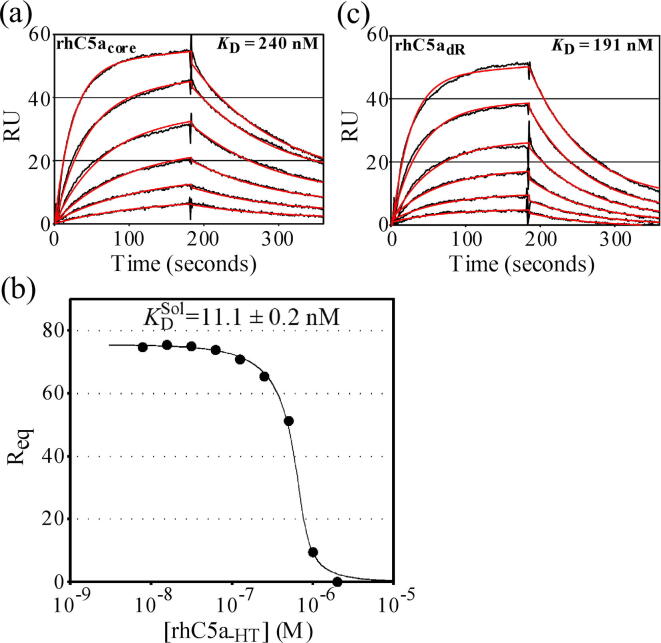
ScpA_S512A_ binding to rhC5a_core_, rhC5a_dR_ and competition binding assay. Panels (a) and (c) show representative sensorgrams of ScpA_S512A_ binding to rhC5a_core_ and rhC5a_dR_ respectively. Observed data (black lines) are plotted with curves obtained from global fitting of data with a 1:1 Langmuir model for binding (red lines). Mean *K*_D_ values obtained from 3 experiments are reported. (b) R_eq_ values from competition binding assay measuring ScpA_S512A_ binding to immobilized rhC5a_core_ in the presence of increasing concentrations of rhC5a in solution (rhC5a_-HT_). The concentration of rhC5a_-HT_ is plotted on a log scale. Fitting of the data to Eq. 1 (solid line) provides the equilibrium dissociation constant for ScpA-rhC5a_-HT_ in solution ($K_{D}^{\mathrm{Sol}}$). The value for $K_{D}^{\mathrm{Sol}}$ is reported with the standard error for the fitting. (For interpretation of the references to colour in this figure legend, the reader is referred to the web version of this article.)

Binding of ScpA to the larger core portion of C5a (rhC5a_core_) was also measured by SPR. rhC5a_core_ represents the larger N-terminal product (P_N_) of ScpA inactivation. As with the full length rhC5a, the binding curves are well accounted for with a 1:1 Langmuir binding model supporting a single binding site for the rhC5a_core_. Fig. 3a shows the global fitting of the sensorgrams obtained for binding of rhC5a_core_. The *K*_D_ for rhC5a_core_ binding was observed to be in the nM range (240 nM, Table 1), approximately eight-fold greater than for the full length rhC5a (Section 3.3).

Though it is likely that rhC5a_core_ and rhC5a bind to the same region on ScpA, it is possible that two distinct sites exist for binding of these entities. This was examined in an SPR based competition binding study using a tag-less form rhC5a (rhC5a_-HT_). Fig. 3b shows that binding of ScpA to immobilized rhC5a_core_ was reduced with increasing concentrations of free rhC5a_-HT_ in solution. This indicates that the full length and core portion of C5a compete for a single binding site on ScpA. Fitting of the competitive binding curve to Eq. 1 provides an estimate for solution binding affinity $\left(K_{D}^{\mathrm{Sol}}\right)$. A $K_{D}^{\mathrm{Sol}}$ of 11.1 nM again supports a high affinity interaction between ScpA and C5a in solution.

Additional insight into how ScpA binds C5a was gained by comparing the binding energies for rhC5a and rhC5a_core_. The difference in binding energy (ΔΔG°, Table 1) between rhC5a and rhC5a_core_ was 1.2 kcal/mol. Thus, interactions with rhC5a_core_ account for the majority (88%) of the 10.2 kcal/mol of total binding energy (ΔG°_bind_, Table 1). The remaining 12% of the binding energy is attributable to interactions with the 7 C-terminal tail residues presumably in the substrate binding cleft. According to the published model for C5a binding [44], the core portion of C5a is too large to fit into the active site cleft. Thus, the high affinity binding of rhC5a_core_ along with the competitive binding data indicated that the bulk of substrate interactions occur at a site outside of the ScpA active site (*i.e.* an exosite).

### 3.5 R74 is the dominant residue contributing to the tail interaction.

To examine the contribution of the R74 residue to substrate specificity the interaction of ScpA_S512A_ with rhC5a_dR_ was examined by SPR. ScpA_S512A_ binds to rhC5a_dR_ with a *K*_D_ of 191 nM (Table 1 and Fig. 3c), intermediate in value between the *K*_D_s for rhC5a and rhC5a_core_. This 6-fold increase in *K*_D_ relative to full length rhC5a indicates that interactions with R74 contributes 1.0 kcal/mol to binding. Thus, interactions with R74 provide the majority (83%) of the 1.2 kcal/mol contributed by interactions with all 7 C-terminal residues.

### 3.6 The 7 C-terminal tail residues facilitate substrate binding.

The SPR experiments also provide rates of association and dissociation for the studied interactions and thus allow further analysis of the substrate binding process. From Table 1, the *k*_a_ for rhC5a is 13.1 X 10^4^ M^−1^s^−1^ while the *k*_a_ values for rhC5a_dR_ and rhC5a_core_ are 6.17 X 10^4^ M^−1^ s^−1^ and 3.52 X 10^4^ M^−1^ s^−1^ respectively. The association rate for binding rhC5a is 2.1 times faster than for rhC5a_dR_ and 3.7 times faster as compared to for rhC5a_core_. Thus, the *k*_a_ for binding decreases when the tail is either removed or truncated, indicating that the presence of the C-terminal tail residues accelerates the association of the substrate. This would suggest that while most of the stabilizing interactions involve the residues in the core of the substrate, the tail residues facilitate the formation of these interactions possibly by aiding proper positioning of the substrate on ScpA. The tail residue interactions in the prime side of the active site are expected to involve the PA domain [44]. Thus, these observations highlight the possibility that interactions between the tail residues and the PA domain facilitate communication between the active site and exosite.

### 3.7 Binding and cleavage of rhC5a to ScpA is dependent on NaCl concentration.

**Fig. 4:**
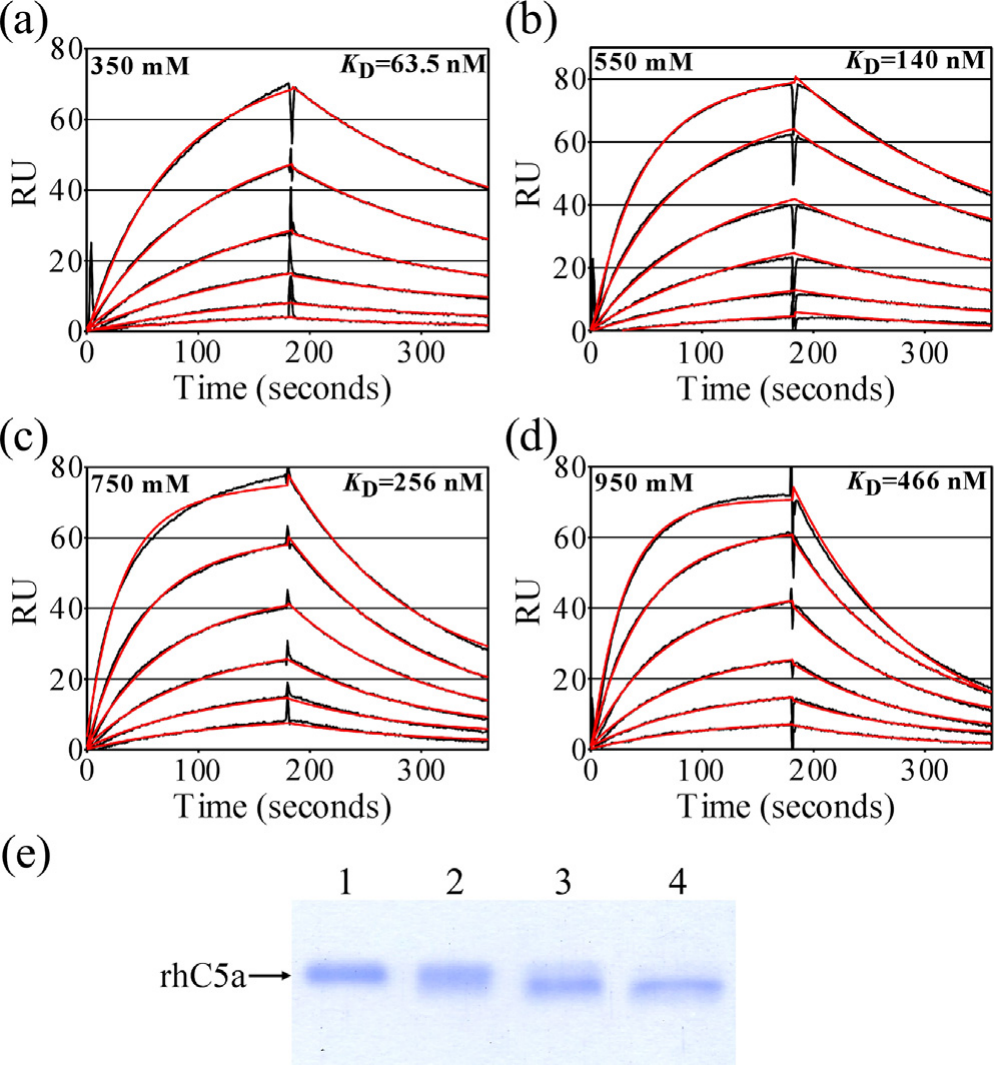
SPR analysis of the salt dependence of ScpA_S512A_-rhC5a binding and activity. Panels (a) through (d) show representative sensorgrams of ScpA_S512A_ binding to rhC5a in the presence of 350, 550, 750 and 950 mM NaCl respectively. Observed data (black lines) are plotted with curves obtained from global fitting of data with a 1:1 Langmuir model for binding (red lines). Mean *K*_D_ values obtained from 3 experiments are reported. (e) ScpA activity against rhC5a in the presence of 10, 150 and 1000 mM NaCl (lanes 2 to 4). Uncleaved rhC5a (lane 1) is shown for comparison. (For interpretation of the references to colour in this figure legend, the reader is referred to the web version of this article.)

Based on amino-acid composition, ScpA and rhC5a are expected to be oppositely charged at pH 7.4 (net charges of −31 and + 5 for ScpA and rhC5a respectively). Thus, rhC5a binding was measured in the presence of increasing NaCl concentrations. Comparison of the sensorgrams for binding at 150 mM (Fig. 2a) versus 350, 550, 750 and 950 mM (Fig. 4a-4d) clearly show the salt dependence of ScpA-rhC5a interaction. The *K*_D_ increased from 34 to 466 nM as buffer NaCl concentrations increased from 150 to 950 mM (Table 1). This decrease in substrate binding affinity is due mainly to a 6.7-fold lower *k*_a_ (13.1 X 10^4^ to 1.953 X 10^4^ M^−1^s^−1^), as compared to an ~ 2-fold increase in *k*_d_ (4.4 X 10^-3^ to 9.10 X 10^-3^ s^−1^). The impact of increased ionic strength on *k*_a_ supports the contribution of electrostatic interactions during the association of the substrate.

In these binding experiments, it is possible that the high concentrations of NaCl may have impacted on the fold of ScpA or possibly the substrate. We therefore evaluated the activity of the active form of ScpA at higher concentrations of NaCl. Interestingly, the simple gel assays show that ScpA not only retains its activity at 1 M NaCl, but in fact cleaves the substrate more efficiently at the higher salt concentration (Fig. 4e). This would not be expected given that substrate affinity decreases with higher salt. The activity assays were conducted with high substrate concentrations (18 µM), possibly well above the *K*_m_ for rhC5a at 1 M NaCl, thus the enhanced activity may be related to a higher V_max_ or turnover rate under these conditions.

### 3.8 Arginine residues, but not lysine residues in the core of C5a contribute to binding

**Fig. 5:**
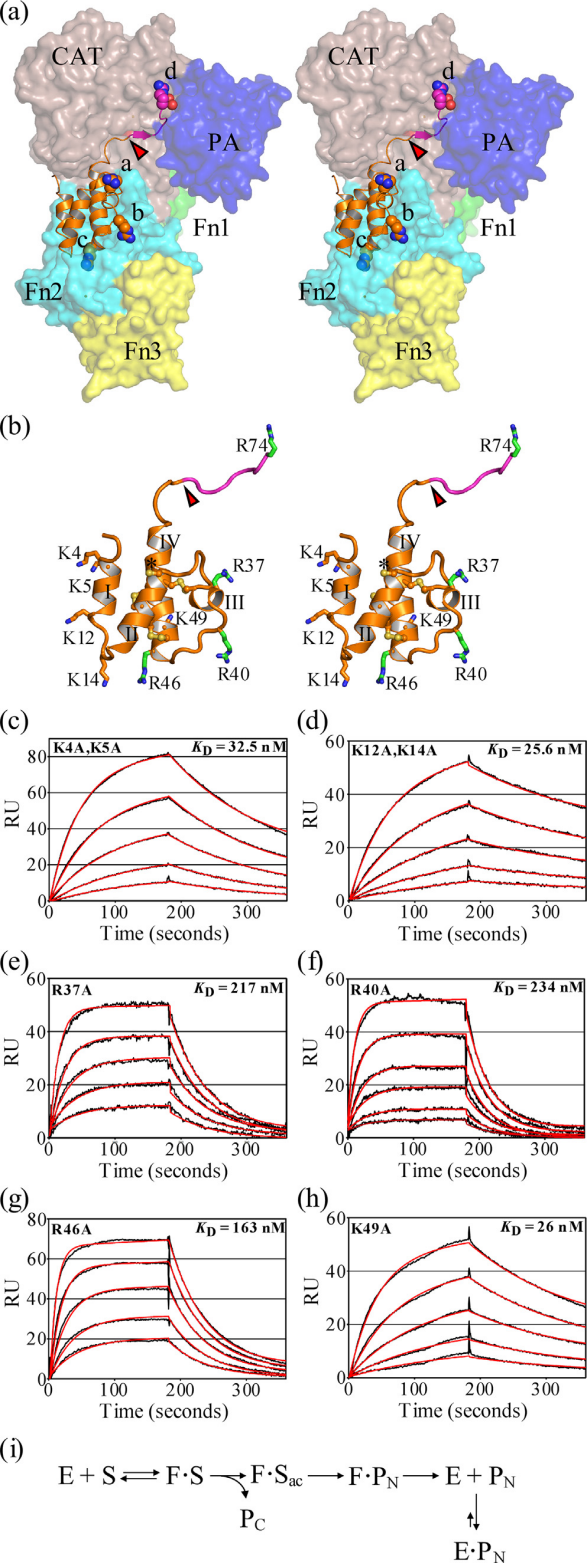
Model of the ScpA-hC5a complex. (a) The stereo diagram shows the ScpA-C5a complex with hC5a (cartoon diagram) on the surface of ScpA. The coordinates for the model are from the docking analysis described in Kagawa *et al.*[44]. ScpA is colored by domains, with the catalytic domain (‘CAT’) domain in salmon, the PA domain in blue, and the Fn1–Fn3 domains in green, cyan, and yellow, respectively. hC5a is shown as a cartoon model with the core portion (P_N_, orange) on the ScpA Fn2 domain and tail residues (P_C_, purple) extended through the prime side of the active site. The location of the scissile bond is indicated with a red arrow. Sidechains of hC5a arginine residues found to impact on binding are shown with space filling models and labelled ‘a’ (R37), ‘b’ (R40), ‘c’ (R46), and ‘d’ (R74). Panel (b) shows a stereo diagram the hC5a model indicating locations of all residues mutated in this study (shown as stick models). hC5a is colored as in panel A. The four hC5a helices are labelled ‘I’ to ‘IV’. The location of the C27 sidechain is indicated with an asterisk. Panels (c) to (h) show representative sensorgrams of ScpA_S512A_ binding to rhC5a_K4A,K5A_, rhC5a_K12A,K14A_, rhC5a_R37A_, rhC5a_R40A_, rhC5a_R46A_, and rhC5a_K49A_ respectively. Observed data (black lines) are plotted with curves obtained from global fitting of data with a 1:1 Langmuir model for binding (red lines). Mean *K*_D_ values obtained from 3 experiments are reported. Panel (i) shows a reaction scheme where binding of full-length hC5a (‘S’) occurs with a conformational change in ScpA (‘E’ to ‘F’). The C-terminal ‘tail’ residues (‘P_C_’) are released during the acylation step. Deacylation produces a complex (‘F∙P_N_’) between the enzyme and core portion of hC5a (‘P_N_’). Dissociation of P_N_ is faster from ‘F’ and thus not rate limiting in the catalytic cycle. Binding of the ‘P_N_’ product to the ‘E’ ScpA state is accompanied by a slow rate of dissociation as measured in SPR studies. (For interpretation of the references to colour in this figure legend, the reader is referred to the web version of this article.)

The binding studies in the presence of higher salt concentrations indicated that association of the substrate involved ionic interactions (Section 3.7). Substrate binding was also shown to be dominated by interactions with rhC5a_core_ (Section 3.4), thus the contribution of positively charged core residues was examined by SPR. A panel of single and double mutants of rhC5a were generated using the published model of the ScpA-C5a complex as a guide (Fig. 5a and 5b show stereo representations of the ScpA-complex and C5a, respectively). ScpA activity was demonstrated against all mutants (Supplemental Fig. S2) supporting proper folding of the substrate.

Mutations in 3 of the 4 total R residues in the C5a core (R37A, R40A and R46A) were observed to decrease binding affinity relative to rhC5a (Table 1). These residues occur in the region encompassing C5a helix III and the loop linking helix III and helix IV (Fig. 5b). The observed *K*_D_s for rhC5a_R37A_, rhC5a_R40A_ and the rhC5a_R46A_ are 217 nM, 234 nM and 162 nM, respectively (Fig. 5e-g), each mutant decreasing the binding energy by approximately 1 kcal/mol. In these arginine mutants, the decrease in affinity is predominantly due to a faster dissociation rates for binding rather than slowing the rate of association. The *k*_d_ for rhC5a_R37A_ (17.6 × 10^-3^ s^−1^), rhC5a_R40A_ (20.5 × 10^-3^ s^−1^) and rhC5a_R46A_ (17.8 × 10^-3^ s^−1^) mutants increased by 4.0, 4.7 and 4.0-fold, respectively, in comparison to rhC5a interaction (4.4 × 10^-3^ s^−1^). This suggests that these residues mainly contribute short range interactions in the bound state rather than facilitating formation of the complex. Of the 3 tested arginine residues in the core, R37 and R40 make the largest contribution to binding impacting both *k*_a_ and *k*_d_ indicating their roles in the formation as well as the stability of the bound state.

Five of the 7 total lysine residues in the C5a core were also mutated to examine their role in binding (Fig. 5b). 4 of the 5 residues (K4, K5, K12 and K14) are in helix I and inter α-helical loop 1, outside the 3 central disulfide bridged helices (helices II-IV). The fifth lysine residue (K49) is in helix IV. These mutations did not affect binding affinity as significantly as mutations of the arginine residues.

The observed *K*_D_s range between 25.6 and 32.5 nM (Fig. 5c, 5d, 5 h and Table 1). Interestingly, the association rates for all lysine mutants (15.8 – 19.5 X 10^4^ M^−1^s^−1^), are marginally faster than for rhC5a (13.1 X 10^4^ M^−1^s^−1^), while the dissociation rates are relatively unchanged. This shows that in contrast to R37, R40 and R46, these lysine residues do not contribute significantly to the stability of the enzyme-substrate complex.

In summary, positively charged residues important for binding identified in these studies are located on one side of the C5a molecule in the region spanning Helix III, Helix IV and the intervening loop.

## 4 Discussion

The importance of complement in human inflammatory diseases is well established. However, in recent months there is increased awareness of the impact of complement activation in disease because of its role in the progress and severity of COVID-19 infections. A recent study reported that C5a levels in COVID-19 patients correlated with disease severity [1], [24]. Several groups are targeting C5a or its biological receptor C5aR1 using monoclonal antibodies (MAbs) to C5, C5a and C5aR1. These studies are showing promise in late-stage clinical trials [2], [3], [4], [5]. The potential of using an enzyme to target C5a has yet to be explored.

Of particular interest is the use of bacterial proteases which naturally target components of the human immune system and modulate the immune response, a type of immunomodulatory enzyme (IME). The potential of exploitation of IMEs is exemplified by the streptococcal proteases IdeS and ScpA. The former targets IgG and is being developed as a candidate therapeutic in clinical trials [37], while the latter targets C5a and has been proposed as a means for targeting the inflammatory response in sepsis [9]. Intervention with therapeutic proteases has clear advantages of use of MAbs. While both types of biologic can be highly specific, the action of enzymes is catalytic in nature as opposed to stoichiometric for MAbs. Thus, the required therapeutic dose of IMEs could be significantly lower in comparison to MAbs. Minimizing the amount of biologic administered to the patient would reduce associated risks of side-effects, and the ultimate cost of the therapy. To better develop ScpA as a potential therapeutic resource, a more complete understanding of the enzyme is essential. This includes characteristics such as mechanical and chemical stability, engineering to remove functions such as fibronectin binding [38], as well as how the specificity of the enzyme is affected by different environmental conditions. Thus, the first objective of these studies was to obtain sensitive tools to benchmark the activity of ScpA and the interaction with its substrate hC5a.

Enzyme kinetic characterization utilizing a substrate based on the full length hC5a shows that ScpA cleaves C5a with a *K*_m_ of 185 nM and the *k*_cat_ a 0.886 s^−1^ (Section 3.2). In comparison, Anderson and co-workers reported a *K*_m_ of 360 µM and a *k*_cat_ of 1.1 s^−1^ using 16 residue peptide which lacks the bulky N-terminal core portion of C5a [19]. The >1000-fold lower *K*_m_, reported herein, indicates that significant interactions with C5a occur outside the active site thus supporting the importance of exosite interactions in substrate recognition. Significantly, a *K*_m_ in the low nM range agrees well with the physiological concentrations of C5a (12 nM) reported in intra-abdominal infection [22]. The analyses also showed that ScpA is a relatively slow enzyme, with the *k*_cat_ value falling at the lower end of reported values for other multidomain subtilases. For example, reported values of *k*_cat_ for Furin can be more than 100 s^−1^ for substrates with nM *K*_m_s [39], [40]. The low *k*_cat_ for ScpA is potentially due to conformational changes related to the enhanced specificity of the enzyme.

The mode of interaction between ScpA and C5a was also examined in SPR binding experiments (Section 3.3). A *K*_D_ of 34 nM was observed for binding between the ScpA_S512A_ inactive mutant and the substrate rhC5a. This is in good agreement with the $K_{D}^{\mathrm{ITC}}$ measured in ITC experiments for solution binding (30.8 nM). The ITC data also showed that binding is enthalpically driven, consistent with the large number of hydrogen bonds and salt-bridges predicted for the complex [44]. The observed binding affinity is much lower than previously reported by Terao and co-workers [21] in studies with the active enzyme (*K*_D_ 7.25 mM). As with the enzyme kinetic studies, the low nM range *K*_D_, reported herein, is in good agreement with what is known about C5a levels *in vivo*. The tools used in these analyses allow probing of ScpA activity and substrate interactions in a manner not previously possible. The methods described here are currently being applied to examine the selectivity of the enzyme for C5a and C3a from different species which will inform interpretation of animal studies on *S. pyogenes* virulence as well as model selection for preclinical evaluation of an ScpA therapeutic.

The SPR experiments (3.3, 3.4, 3.7, 3.8, 3.7, 3.8) with the full length rhC5a and truncated forms of rhC5a (rhC5a_core_ and rhC5a_dR_) support 4 general features of the proposed model for substrate binding by ScpA [44]. First, rhC5a binding by ScpA is dominated by interactions between the core portion of rhC5a and an exosite, which we propose to be located on the Fn2 domain due to its proximity to the entrance of the active site. Participation of an exosite is indicated by the nM binding affinity of rhC5a_core_ (*K*_D_ 240 nM) accounting for 88% of the substrate binding energy (ΔG°_bind_). Residues of the rhC5a_core_ form an α-helical bundle, reported to be too bulky to access the ScpA active site in docking studies [44]. Second, interactions with the 7 C-ter C5a tail residues make a smaller contribution to substrate binding affinity. Observed binding affinities for rhC5a_core_ and rhC5a_dR_ show that interactions with the seven C5a tail residues (68–74) contribute 12% or 1.2 kcal/mol of ΔG°_bind_ of which 83% or 1 kcal/mol involve hC5a residue R74. The tail residues are the C-ter (P_C_) product released by ScpA and thus expected to bind in the prime side of the active site in an extended conformation with potential interactions involving residues of the catalytic and PA domains [44]. Interestingly, the presence of the tail residues was also shown to increase the rate of association. Thus, interactions between the tail residues and the PA domain may play a role in communicating events in the active site to the exosite and/or participate in orienting the substrate during binding. The third feature supported in these rhC5a binding experiments is the participation of electrostatic interactions in substrate binding. Point mutations, removing a subset of positively charged side chains in the substrate, impacted on substrate binding. In addition, the binding affinity for rhC5a was observed to decrease by nearly 14-fold as the concentration of NaCl increased from 150 mM to 950 mM. The decrease in affinity is mainly a result of a 6.7-fold decrease in the *k*_a_. The slower association rates at higher NaCl concentrations would be consistent with shielding of longer-range interactions between the large lobes of oppositely charged electrostatic potential located on the hC5a core and ScpA Fn2 domain. Finally, these features are consistent with the proposed two-step binding model where electrostatic recruitment of the substrate to the ScpA surface, combines with conformational changes in governing substrate specificity. This is supported by the magnitude of *k*_a_ for rhC5a, which is in a range consistent with protein interactions that are governed neither by diffusion nor by slower large-scale conformational changes [36].

The SPR studies also examined binding to a panel of hC5a mutants to gain additional insight into ScpA substrate specificity. Alanine mutants of 8 positively charged K and R residues were tested since ScpA and hC5a are oppositely charged at neutral pH and substrate binding affinity was observed to be salt dependent (Section 3.7). Mutation of 3 R residues in Helix III (R37), Helix IV (R46) and in the Helix III-IV loop (R40), which are in the cross-linked C5a core, each decreased binding affinity by approximately 1 kcal/mol. The impact of these mutations on substrate binding affinity provides additional evidence that exosite interactions play a significant role in hC5a binding. Interactions with these residues would involve residues outside of the ScpA active site since they are located more than 20 Å from the scissile bond. In contrast, mutation of the 5 K residues (Fig. 5c, 5d and 5h) in Helix I (K4, K5 and K12), Helix IV (K49) and in the Helix I-II loop of C5a (K14) had little effect on the binding affinity. These residues do not form specific interactions with ScpA since the mutations did not significantly alter the *k*_d_ of binding. The expectation based on the high salt binding studies was that mutations of lysine or arginine residues would identify residues involved in long range electrostatic interactions. However, no single mutation accounts for the observed decrease in *k*_a_ at high salt. It is possible that the long-range effect involves other untested charged residues and/or is a cumulative effect over multiple residues.

While the data presented here support the general features of the model of the ScpA-C5a complex proposed [44], discrepancies between the kinetic parameters obtained from the enzymatic and SPR analyses may indicate additional complexity in the ScpA catalytic cycle. It is often assumed that proteases follow the 3-step mechanism for substrate hydrolysis. This involves binding of the substrate to form the Michaelis complex, followed by two chemical transformation steps (acylation and deacylation). The rate of dissociation for the second product (P_N_) is typically thought to be fast relative to the rate of deacylation and thus not explicitly included in the expressions for *k*_cat_ or *K*_m_. For ScpA, dissociation of the core portion (P_N_) measured by SPR is very slow (*k*_d_ 8.5 X 10^-3^ *sec*^-1^ for rhC5a_core_) and would be rate limiting for a subtilase [39]. However, the rate of substrate hydrolysis by ScpA is clearly not limited by the P_N_ dissociation rate measured by SPR with a more than 100-fold faster *k*_cat_ (0.886 *sec*^-1^). The inconsistencies in these values may indicate that efficient progression through the catalytic cycle involves ScpA conformational state(s) that require appropriate interactions with both core and tail residues in an intact full-length substrate. This is illustrated in Fig. 5i where an ScpA conformational state ‘F’ is adopted in the ES complex upon binding of the full length hC5a. Release of the core in the catalytic cycle would occur from a conformational state accessed only in the presence of the tail. Dissociation of the P_N_ from this state may be faster than indicated by SPR studies, and thus not rate limiting. In the absence of the tail residues, the core binds to ScpA with a slow dissociation rate potentially forming an inhibited enzyme complex. This scenario implies that recognition of both parts of substrate are critical for a productive encounter with the enzyme and may be an important feature in the specificity of ScpA and similar enzymes.

The emerging view of ScpA and related subtilases is that selection of their substrates is a dynamic two-step process involving flexibility in the domains around the active site and in the C-ter of the substrate. Structural and molecular dynamics studies indicate that the PA domains in ScpA and the related IL-8 cleaving subtilisin SpyCEP are capable of adopting different orientations [41]. In mutagenesis studies, Bruinenberg et al. demonstrated that deletion of the PA domain in the CEP from *Lactococcus lactis* modified the caseinolytic specificity but not the activity of the enzyme [42]. As suggested for SpyCEP recognition of the N-terminal ELR motif in IL-8, mobility of the PA domain in ScpA could allow crucial interactions with the N-terminal core portion of the C5a as well as with the Fn2 domain and thus either open or modify features in the prime side of the active site. In this manner, PA domain sensing of appropriate contacts at the exosite would be an additional mechanism for ensuring proper substrate selection. Additional studies with ScpA mutants are required to unequivocally show that residues distant from the active site, for example in the Fn2 domain, are important for substrate binding and specificity. Using the tools developed herein, it is possible to evaluate these mutations systematically. Furthermore, it is possible to test the susceptibility of C5a from different species to the action of ScpA, which will contribute to a better evaluation of pre-clinical animal models.

## 5 Concluding remarks

The enzymatic and binding studies presented here provide the first detailed information on the activity and specificity of the C5a peptidase ScpA. Bioinformatic analysis identified many CEPs in Gram positive organisms that inhabit the human gut [43] which may participate in modulating the host immune response in a manner similar to ScpA. This makes ScpA the prototype of an expanding group of immuno-modulatory enzymes (IMEs) with potential medical and biotechnological applications. The use of IMEs as a strategy for therapeutic intervention has clear advantages over use of MAbs. Both can be highly specific, but the catalytic nature of enzymes means that the dose of therapeutic required for treatment would be significantly lower for an enzyme. Minimizing the amount of biologic administered to the patient reduces associated risks of side-effects, and the ultimate cost of the therapy. Engineering of IMEs, such as ScpA, for exploitation as anti-complement or anti-cytokine therapies will require detailed analyses, such as those reported here, to establish substrate preference or hierarchy of activity against these important immune modulators.

## CRediT authorship contribution statement

**Malgorzata Teçza:** Investigation, Methodology, Formal analysis, Writing - original draft. **Todd F. Kagawa:** Investigation, Methodology, Conceptualization, Formal analysis, Writing - original draft, Writing - review & editing. **Monica Jain:** Investigation, Writing - original draft. **Jakki C. Cooney:** Investigation, Conceptualization, Writing - original draft, Writing - review & editing, Funding acquisition, Supervision.

## Declaration of Competing Interest

The authors declare the following financial interests/personal relationships which may be considered as potential competing interests: JCC and TFK are inventors on a patent for the development of ScpA as a therapeutic for sepsis, licenced to a company through the University of Limerick.
