# Supplementary material for: Enzyme kinetic and binding studies identify determinants of specificity for the immunomodulatory enzyme ScpA, a C5a inactivating bacterial protease
Source: Comput Struct Biotechnol J. 2021 Apr 17;19:2356–65. doi: 10.1016/j.csbj.2021.04.024 (PMC8052502; doi:10.1016/j.csbj.2021.04.024)
Supplement: Supplementary data 1 [file mmc1.docx]

**Supplemental information**

**SI1. Preparation of Recombinant Proteins**

The cloning of recombinant ScpA has been described previously [1]. Briefly, the C5a peptidase gene (coding amino-acid residues A31-S1032) was PCR amplified from the genome of *S. pyogenes* B220, and cloned into the pGEX-6P-3 expression vector (GE Healthcare, UK), generating pGEX ScpA_(31-1032)_. For the purposes of this work the expressed protein is called ScpA. The active site serine (S512) was mutated to an alanine residue with the QuickChange II site directed mutagenesis kit (Strategene, USA) using pGEX ScpA_(31-1032)_ as a template. The glutathione S-transferase (GST) tagged ScpA and S512A mutant ScpA (ScpA_S512A_) proteins were over-expressed in *Escherichia coli* DH5α (Invitrogen, UK). Purification of ScpA_S512A_ followed a similar protocol established for ScpA [1]. All affinity chromatography steps (chelating-Sepharose and glutathione-Sepharose 4B, GE Healthcare, UK) were run with a Bio-Rad Econo System (Bio-Rad, USA). Additional purification with anion-exchange (HiTrap Q HP, GE Healthcare, UK), cation-exchange (UNO-S, Bio-Rad, USA) or size exclusion chromatography utilized an Äkta Prime Plus FPLC system (GE Healthcare, UK). GST-tagged ScpA_S512A_ was initially affinity purified by passing cleared cell lysates through a Glutathione-Sepharose 4B column. The resin bound protein was washed with phosphate buffer saline (PBS, 140 mM NaCl, 2.7 mM KCl, 10 mM Na_2_HPO_4_, 1.8 mM KH_2_PO_4_) and eluted with 10 mM reduced glutathione in PBS buffer. The propeptide and N-terminal tag of ScpA_S512A_ was removed by SpeB, the cysteine protease from *S. pyogenes*. The processing reaction followed the protocol described by Anderson *et al*. in which limited proteolysis by SpeB was reported to produce the mature form of ScpA with an N-terminal K90 residue [2]. 7.7 μM of mutant ScpA was treated with 1.8 μM SpeB purified from culture supernatants as described by Kagawa *et al.* [3]. The reactions were conducted at 25 °C in PBS with 20 mM DTT and stopped after 10 minutes by addition 100 μM iodoacetamide (Sigma Aldrich, USA). The reaction mixtures were separated with anion exchange chromatography (HiTrap Q HP) using a linear NaCl gradient (10 to 1000 mM) in 10 mM Tris-HCl pH 8. Any uncleaved tagged peptidase or remaining GST tag was removed with affinity chromatography (glutathione-Sepharose 4B).  Gel filtration (Superdex 200, GE Healthcare, UK) was used to exchange the sample into 50 mM Hepes-KOH pH 7.5, 100 mM NaCl, 0.01% sodium azide. The purified ScpA proteins were stored in small aliquots at -80 °C. The proper folding of ScpA_S512A_ was confirmed crystallographically (Section SI4).

Recombinant human C5a peptides (rhC5a, rhC5a_dR_ and rhC5a_C75_) were produced as N-ter hexa-histidine tagged (HT) fusion proteins using a protocol which closely followed the method of Bubeck *et al.* [4]. Synthetic genes for human C5a were inserted into the pProExHTb expression vector (Invitrogen, UK). Unless otherwise stated point mutations in C5a were generated using the QuikChange II site directed mutagenesis kit (Stratagene, USA) with specific primers, and using the pProEXHTb construct for rhC5a as the template. In each case the targeted amino acid (K4, K5, K12, K14, R37, R40, R46, and K49) was substituted by an Ala residue. The synthetic gene for rhC5a_C75_ was generated using *gblock* technology (IDT Biotech) and cloned into pProEXHTb for expression.

All HT-rhC5a fusion proteins were over-expressed as inclusion bodies in *Escherichia coli* DH5α, and refolded under reducing conditions. The cell paste harvested from 2 L of culture was dissolved in 40 mL of 6 M guanidine thiocyanate and flash diluted into 1 L of refolding buffer (50 mM Tris-HCl pH 8, with 5 mM β-mercaptoethanol) and stirred overnight at room temperature. The precipitated cell components were removed with centrifugation, and the soluble proteins separated with affinity (Nickel-chelating-Sepharose), and cation exchange chromatography (UNO-S) using a linear NaCl gradient (0-2 M NaCl, 10 mM sodium acetate, **
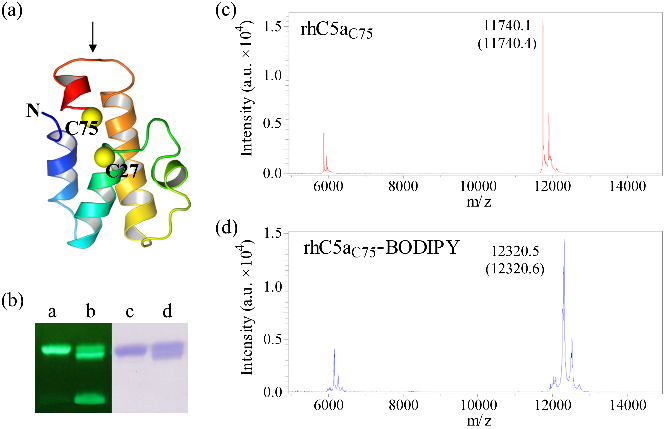
**pH 5). Gel filtration (Superdex 75, Amersham Biosciences, USA) was used to exchange the peptide samples into 50 mM Hepes-KOH pH 7.5, 50 mM NaCl.

The core fragment of recombinant human C5a (rhC5a_core_), was produced by incubating the purified rhC5a and ScpA samples as described in Kagawa *et al.* [1]. Reactions were conducted at 20 °C in 50 mM Hepes-KOH pH 7.5, 100 mM NaCl. Based on SDS-PAGE of reaction time points, rhC5a was completely converted to rhC5a_core_ within 60 minutes. Mass spectrometry of rhC5a_core_ indicated that treatment with ScpA results in the cleavage of rhC5a between H67 and K68. The rhC5a_core_ product was further purified with affinity chromatography (Nickel-chelating-Sepharose) and dialysed into 50 mM Hepes-KOH pH 7.5, 50 mM NaCl. All purified C5a peptides migrated as a single band on non-reducing and reducing SDS-PAGE gels and were confirmed to be mainly α-helical with CD spectropolarimetry (Chirascan, Applied Photophysics) (data not shown). The purified recombinant peptides were stored in aliquots at -80 °C.

For the competition binding assay, the N-ter HT was removed from rhC5a using TEV protease (Sigma Merck, U.S.A). Briefly, 110 µM of rhC5a in 1.3 mL of 25 mM Tris-HCl, pH 8.0, 50 mM NaCl is cleaved with 200 U of TEV protease at 4 °C for 24 hours. The cleaved product (rhC5a_-HT_) is separated from rhC5a and the TEV protease with affinity chromatography (Nickel-chelating-Sepharose) and exchanged into 10 mM Hepes-KOH pH 7.5, 150 mM NaCl for surface plasmon resonance (SPR) experiments.

**SI2. Production and labelling of rhC5a_C75_-BODIPY**

rhC5a_C75_ was fluorescently labelled with BODIPY™ FL Iodoacetamide (Invitrogen, U.S.A) for the enzyme kinetic assays. BODIPY™ FL dye groups were introduced on either side of the scissile bond at residues C27 and C75 in the rhC5a_C75_ mutant (Fig. S1a). Purified rhC5a_C75_ (50 µM) in 100 mM

**Fig. S1 Supporting data for BODIPY FL labelling of rhC5a_C75_.** (**a**) Cartoon diagram hC5a illustrating locations of the naturally occurring C27 and introduced C75 residues targeted by the labelling reaction. The location of C75 has been modelled using the NMR structure of human C5a (PDB index 1KJS) [5]. The location of the scissile bond is indicated with an arrow. (**b**) SDS-PAGE analysis of rhC5a_C75_-BODIPY cleavage by ScpA. Lanes ‘a’ and ‘b’ are visualized by fluorescence while proteins have been stained with Coomassie in lanes ‘c’ and ‘d’. Lanes ‘a’ and ‘c’ show rhC5a_C75_-BODIPY and lanes ‘b’ and ‘d’ show rhC5a_C75_-BODIPY treated with ScpA. Two fluorescent bands are produced by ScpA cleavage of rhC5a_C75_-BODIPY, consistent with the incorporation of BODIPY FL in the tail and core of the substrate. Panels **c** and **d** show MS data for unlabelled and labelled rhC5a_C75_, respectively. Observed and calculated (in parenthesis) masses are reported. An increase in mass of 289.1 Da is expected for each BODIPY FL molecule. A difference of 580.5 Da between the labelled and unlabelled proteins is consistent with two BODIPY FL moieties in rhC5a_C75_-BODIPY.

Tris/HCl pH 8, 150 mM NaCl was first reduced by addition of Tris(2-carboxyethyl)phosphine hydrochloride solution (TCEP) to a final concentration of 2 mM with agitation at room temperature for 20 min. 0.1 M BODIPY™ FL Iodoacetamide in anhydrous DMSO (Sigma Merck, U.S.A) was added to the reduced protein to a final concentration of 2 mM. The labelling reaction was conducted at room temperature for 24 hours. The labelled protein was exchanged into PBS buffer and separated from the free label using a PD-10 column (GE Healthcare, UK). Protein concentration was determined following a correction for the
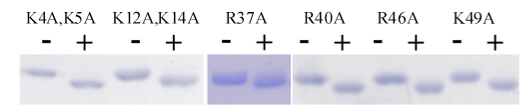
BODIPY absorbance at 280 nm. The doubly labelled substrate is readily cleaved by ScpA (Fig. S1a). Label incorporation and ScpA cleavage products were confirmed with mass spectrometry (MS) (Fig. S1c-d).

**SI3. The activity assays for ScpA on rhC5a mutants**

The activity assays for ScpA on rhC5a mutants were performed in 50 mM Hepes/KOH pH 7.4, 100 mM NaCl. 40 μM of each rhC5a mutant was incubated with 40 nM of ScpA at room temperature 30 min. The reaction was terminated by adding SDS-PAGE sample loading buffer and heating at 95 °C for 5 minutes. Reaction were analyzed by SDS-PAGE[6]. A drop in molecular weight was observed for all mutants following treatment with ScpA (Fig. S2). Western blots with antibodies targeting the N-terminal His-tag confirmed that ScpA cleaved the rhC5a mutants at the C-terminus (data not shown). The loss of the 7 C-terminal tail residues was confirmed by MS for rhC5a_R37A_, rhC5a_R40A_ and rhC5a_R46A_ cleavage products (Table S1).

**Fig. S2** Cleavage of rhC5a mutants by ScpA. SDS-PAGE analysis of rhC5a_K4A,K5A_, rhC5a_K12A,K14A_, rhC5a_R37A_, rhC5a_R40A_, rhC5a_R46A_, rhC5a_K49A_ cleaved by ScpA. Lanes labelled ‘+’ and ‘–’ indicate samples treated or not treated with


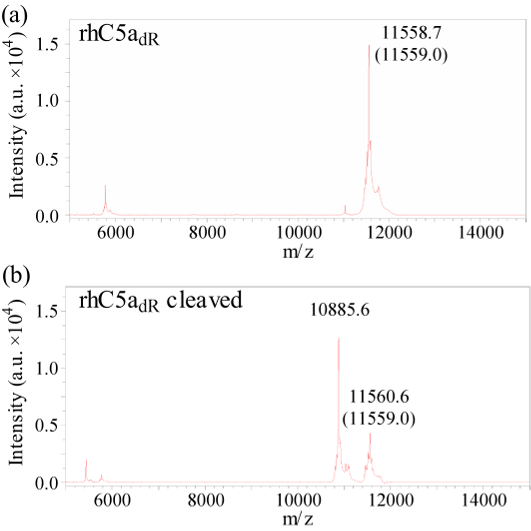
ScpA, respectively

| **Table S1. Mass spectrometry of cleaved rhC5a mutants** | |
| --- | --- |
| Sample | Mass*^1^* |
| rhC5a_R37A_ | 11624.4 (11630) |
| rhC5a_R37A_ cleaved | 10804.2 (10801) |
| rhC5a_R40A_ | 11629.1 (11630) |
| rhC5a_R40A_ cleaved | 10803.2 (10801) |
| rhC5a_R46A_ | 11623.3 (11630) |
| rhC5a_R46A_ cleaved | 10800.5 (10801) |
| ^1^ Observed and calculated (in parenthesis) masses are reported in Da. | |

The ability of ScpA to cleave rhC5a_dR_ was examined in similar assays. 18 μM rhC5a_dR_ was treated with 10 nM ScpA in PBS and incubated at 37 °C for 15 minutes. Cleavage products were assessed with SDS-PAGE and MS (Fig S3).

**Fig. S3 Mass spectrometry of rhC5a_dR_ cleaved by ScpA.** Panels (**a**) and (**b**) show MS data for rhC5a_dR_ and rhC5a_dR_ treated with ScpA. Observed and calculated (in parenthesis) masses of rhC5a_dR_ are shown in both panels. Panel (**b**) shows that cleaved rhC5a_dR_ has an observed mass of 10885.6 Da, consistent with rhC5a_core_ (Fig. 1a and 1b) and the loss of the 6 rhC5a_dR_ C-ter residues.

**SI4. Crystallographic structure solution of ScpA_S512A_**

The fold of the S512A ScpA active site mutant (ScpA_S512A_) was confirmed by X-ray crystallography. Following purification and processing with SpeB, ScpA_S512A_ was exchanged into 50 mM Hepes/KOH (pH 7.5) and 100 mM NaCl buffer with size exclusion chromatography. The peak fractions were pooled and concentrated to 11.0 mg/mL. The protein crystallization samples were aliquoted and stored at −80 °C. Crystals were grown using the hanging-drop vapor-diffusion method at room temperature, by mixing 1 μL of protein solution and 1 μL of reservoir solution composed of 2.0 M ammonium sulfate and 0.2 M Hepes/KOH pH 7.5. Crystals were flash-cooled under liquid nitrogen to 110 K in 2.0 M ammonium sulfate, 0.2 M Hepes/KOH pH 7.5, and 0.8 M sodium malonate cryoprotectant.

X-ray diffraction data were collected at a wavelength of 1.5418 Å using a Rigaku MicroMax-007 X-ray source with a Rigaku R-AXIS IV++ image-plate detector. ScpA_S512A_ crystallized in the same space group as the active form of the enzyme (*P*6_3_22) with unit cell dimensions of a=167.4 Å, b=167.4 Å, and c=141.8 Å, with one protein molecule per asymmetric unit. The data were processed to 2.6 Å resolution with MOSFLM [7] and AIMLESS [8] of the CCP4 suite [9]. Statistics for data collection and processing are reported in Table S2.

A molecular replacement solution was obtained with PHASER [10] using the backbone atoms from the structure of ScpA (PDB code 3EIF) as search model [1]. The remaining structure was built in Coot and refined with PHENIX. The final model of ScpA_S512A_ includes residues 97–1032 with a single bound calcium ion. The R-work and R-free values for the final model are 20.8% and 26.4%, respectively. Other statistics pertinent to data collection and final refinement are given in Table S2. The quality of the model was assessed with MOLPROBITY [12].

The density of the catalytic triad residues D130 and H193 as well as the Ala mutation at residue 512 are well accounted for in the electron density map (Fig. S4a and b). The structure of ScpA_S512A_ is nearly identical to the active form with an overall RMSD for 934 Cα carbons of 0.2 Å and low residue RMSD values between the two structures (Fig. S4c). A larger difference is observed for residue D804 located in a loop with higher temperature factors. The coordinates and structure factors have been deposited in the Protein Data Bank with accession code 7BJ3.


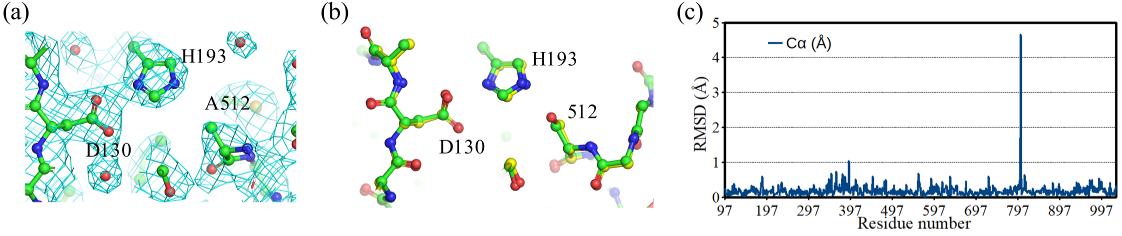


**Fig. S4** Comparison of ScpA_S512A_ and ScpA crystal structures. (**a**) Electron density map showing active site residues D130, H193 and mutated residue A512. (**b**) Superposition of ScpA (yellow carbon atoms) and ScpA_S512A_ (green carbon atoms) active sites. (**c**) Plot of RMSD between Cα carbons of ScpA and ScpA_S512A_ residues. An overall RMSD of 0.2 Å (934 Cα) was observed between the structures.

| **Table S2. Data collection and refinement parameters** | |
| --- | --- |
| *Data collection^a,c,d^* |  |
| Space group | *P*6_3_22 |
| Unit cell dimensions *a, b, c* (Å) | 167.4, 167.4, 141.8 |
| Unit cell dimensions α, β, γ (°) | 90 90 120 |
| Wavelength (Å) | 1.5418 |
| Resolution range (Å) | 36.04 - 2.6 |
| Completeness (%) | 100.0 (100.0) |
| Total reflections | 203030 (24350) |
| Unique reflections | 36528 (3572) |
| Multiplicity | 5.6 (5.6) |
| *R_pim_*/*R_meas_* | 0.125 (0.509) /0.299 (1.213) |
| Mean I/sigma(I) | 7.5 (1.6) |
| Wilson B-factor (Å^2^) | 19.8 |
| Molecules per asymmetric unit | 1 |
|  |  |
| *Refinement statistics^b^* |  |
| Reflections used in refinement | 36524 (2636) |
| Reflections used for *R-free* (5% of total) | 1825 (133) |
| *R-work*/*R-free* | 0.2063 (0.2988)/0.2646 (0.3807) |
| Number of non-hydrogen protein atoms | 6998 |
| Number of non-hydrogen ligand/solvent atoms | 79/184 |
| Protein residues | 936 |
|  |  |
| Ramachandran favored, allowed, outliers (%) | 95.50,4.50, 0 |
| RMS bonds (Å)/RMS angles (°) | 0.002/0.52 |
| Rotamer outliers (%) | 0.85 |
| Clashscore | 3.08 |
| Average B-factor (overall) (Å^2^) | 24.89 |
| protein, ligands, solvent | 24.77, 48.06, 19.48 |
| a Data collection values in parenthesis are for highest resolution shell (2.72 - 2.60 Å). | |
| b Refinement values in parenthesis are for highest resolution shell (2.67 - 2.60 Å). | |
| c *R_pim_*= precision-indicating merging R-factor=Σ*_hkl_*[1/(N-1)]^1/2^ Σ*_i_*\|I*_i_*(*hkl*)-〈I*_i_*(*hkl*)〉\|/Σ*_hkl_*Σ*_i_*I*_i_*(*hkl*). | |
| d *R_meas_*= redundancy-independent merging R-factor=Σ*_hkl_*[N/(N-1)]^1/2^Σ*_i_*\|I*_i_*(*hkl*)-〈I*_i_*(*hkl*)〉\|/Σ*_hkl_*Σ*_i_*I*_i_*(*hkl*). | |

**References**

1. Kagawa, T.F., et al., *Model for substrate interactions in C5a peptidase from Streptococcus pyogenes: A 1.9 Å crystal structure of the active form of ScpA.* J Mol Biol, 2009. **386**(3): p. 754-72.

2. Anderson, E.T., et al., *Processing, stability, and kinetic parameters of C5a peptidase from Streptococcus pyogenes.* Eur J Biochem, 2002. **269**(19): p. 4839-51.

3. Kagawa, T.F., W. O'Toole P, and J.C. Cooney, *SpeB-Spi: a novel protease-inhibitor pair from Streptococcus pyogenes.* Mol Microbiol, 2005. **57**(3): p. 650-66.

4. Bubeck, P., et al., *Site-specific mutagenesis of residues in the human C5a anaphylatoxin which are involved in possible interaction with the C5a receptor.* Eur J Biochem, 1994. **219**(3): p. 897-904.

5. Zhang, X., et al., *Structural definition of the C5a C terminus by two-dimensional nuclear magnetic resonance spectroscopy.* Proteins, 1997. **28**(2): p. 261-7.

6. Laemmli, U.K., *Cleavage of structural proteins during the assembly of the head of bacteriophage T4.* Nature, 1970. **227**(5259): p. 680-5.

7. Battye, T.G., et al., *iMOSFLM: a new graphical interface for diffraction-image processing with MOSFLM.* Acta Crystallogr D Biol Crystallogr, 2011. **67**(Pt 4): p. 271-81.

8. Evans, P.R. and G.N. Murshudov, *How good are my data and what is the resolution?* Acta Crystallogr D Biol Crystallogr, 2013. **69**(Pt 7): p. 1204-14.

9. Winn, M.D., et al., *Overview of the CCP4 suite and current developments.* Acta Crystallogr D Biol Crystallogr, 2011. **67**(Pt 4): p. 235-42.

10. McCoy, A.J., et al., *Phaser crystallographic software.* J Appl Crystallogr, 2007. **40**(Pt 4): p. 658-674.

11. Liebschner, D., et al., *Macromolecular structure determination using X-rays, neutrons and electrons: recent developments in Phenix.* Acta Crystallogr D Struct Biol, 2019. **75**(Pt 10): p. 861-877.

12. Chen, V.B., et al., *MolProbity: all-atom structure validation for macromolecular crystallography.* Acta Crystallogr D Biol Crystallogr, 2010. **66**(Pt 1): p. 12-21.
